# Genome sequences of five phylogenetically diverse bacterial isolates from the *Populus* root microbiome

**DOI:** 10.1128/mra.00851-26

**Published:** 2026-09-11

**Authors:** Delaney G. Beals, Zachary Crockett, Dinh An Phan, An Nguyen, Christopher W. Schadt, Robert W. Cottingham, Dale A. Pelletier

**Affiliations:** 1 Biosciences Division, Oak Ridge National Laboratory 6146, Oak Ridge, Tennessee, USA; USDA-ARS San Joaquin Valley Agricultural Sciences Center, Parlier, California, USA

**Keywords:** plant-microbe interactions, poplar, endosphere, rhizosphere-inhabiting microbes

## Abstract

Five bacterial genomes were sequenced from isolates obtained from *Populus deltoides* and *Populus trichocarpa* roots collected in Tennessee and Oregon. These genomes expand the representation of underrepresented bacterial genera within the *Populus* root microbiome and provide new reference resources for studies of plant-associated bacteria.

## ANNOUNCEMENT

We report genome sequences for five *Populus* root-associated bacterial isolates representing underrepresented genera, expanding the phylogenetic breadth of the *Populus* root microbiome. Root samples collected from *Populus* in Tennessee and Oregon between 2010 and 2012 were transported on wet ice for processing. Rhizosphere isolates were obtained from root-associated soil, and endosphere isolates were obtained from surface-sterilized roots using previously described methods (1). Isolates were recovered by dilution plating on Reasoner’s 2A (R2A) agar (BD Difco, Franklin Lakes, NJ, USA) (2) and incubated at 28°C for 2 days. *Frigoribacterium* sp. CF331 was isolated in 2010 from the rhizosphere of *Populus deltoides* in the Caney Fork River basin, Tennessee (36.1000°N, −85.8333°W). In 2012, *Glaciimonas* sp. OK215 (endosphere) and *Dyella* sp. OK584 (rhizosphere) were isolated from *Populus trichocarpa* in Clatskanie, Oregon (46.1007°N, −123.2000°W), while *Frondihabitans* sp. OV104 (rhizosphere) and *Shinella* sp. OV683 (endosphere) were isolated from *P. trichocarpa* roots collected in Corvallis, Oregon (44.5677°N, −123.2760°W).

Taxonomic identification preceded genome sequencing and was performed by amplification of the 16S rRNA gene using primers 27F and 1492R, followed by Sanger sequencing and BLASTn comparison against the NCBI rRNA database. Best-match accession numbers were CF331 (99.35% identity; NR_026511.1), OK215 (98.37%; NR_109670.1), OK584 (98.91%; NR_121724.1), OV104 (98.31%; NR_125644.1), and OV683 (97.59%; NR_181841.1).

Frozen glycerol stocks were revived on R2A agar, and single colonies were grown aerobically in R2A broth at 28°C overnight before genomic DNA extraction using the DNeasy Blood and Tissue Kit (Qiagen); DNA concentration was measured using a Qubit fluorometer (Thermo Fisher Scientific). Sequencing was performed by Plasmidsaurus (Louisville, KY, USA). Libraries were prepared without amplification, shearing, or size selection using the Oxford Nanopore Technologies (ONT) Ligation Sequencing Kit V14 and sequenced on PromethION R10.4.1 flow cells. Basecalling with Dorado v4.3 (ONT) in super-accurate mode using default Q10 filtering yielded 29.3k–681k reads per sample.

Reads were filtered with Filtlong v0.2.1 (3) to remove the lowest-quality 5%. Assemblies were generated with Autocycler v0.5.1 (4) using Flye v2.9.6+ (5), Hifiasm v0.25.0 (6–8), and Plassembler v1.8.0+ (9), followed by contig filtering, consensus selection, rotation with dnaapler v1.2.0 (10), and polishing with Medaka v1.8.0 (ONT). Assembly quality, circularity, and completeness were assessed using QUAST v4.4 (11), Bandage v0.8.1 (12), and CheckM v1.2.2 (13), respectively; default parameters were used except where otherwise noted.

Genome sizes ranged from 3.42 to 6.82 Mbp, with GC contents ranging from 56.4% to 71.8% (Table 1). Final assemblies consisted of one to four circular contigs per genome, with chromosomal coverage ranging from 53.5× to 99.9× and assembly N50 values ranging from 3.34 Mbp to 6.37 Mbp. Genome annotations were generated using the NCBI Prokaryotic Genome Annotation Pipeline (PGAP) v6.11. Based on GTDB-Tk v2.3.2 (14–16) taxonomy and 16S rRNA gene identity, the isolates were classified as *Frigoribacterium* sp. CF331, *Glaciimonas* sp. OK215, *Dyella* sp. OK584, *Frondihabitans* sp. OV104, and *Shinella* sp. OV683. These genomes expand the representation of Actinobacteria and Proteobacteria within the *Populus* root microbiome and provide references for comparative and functional studies of plant-associated bacteria.

**TABLE 1:** Genome statistics for newly sequenced, *Populus* root-associated bacterial isolates

Strain	Contigs (*n*)	Raw reads (*n*)/*rN*50 (bp)	Genome size (bp)	GC (%)	Assembly *N*50 (bp)	Predicted protein- coding genes (n)	Completeness (%)/Contamination (%)	Contig length (bp)/Classification	Contig coverage (×)
*Frigoribacterium* sp. CF331	2	29,315/24,395	3,423,150	71.83	3,337,855	3,118	98.99/0.17	3,337,855/chromosome	53.5
								85,295/plasmid	652.1
*Glaciimonas* sp. OK215	4	681,539/7,181	6,819,233	56.39	6,369,111	6,143	99.44/0.86	6,369,111/chromosome	97.1
								329,092/unnamed contig	238.7
								30,782/unnamed contig	38.6
								90,248/unnamed contig	39.3
*Dyella* sp. OK584	1	182,172/18,104	4,477,970	62.54	4,477,970	3,972	99.66/1.49	4,477,970/chromosome	99.9
*Frondihabitans* sp. OV104	1	53,129/18,855	3,643,546	69.83	3,643,546	3,384	97.98/0.42	3,643,546/chromosome	98.0
*Shinella* sp. OV683	2	91,970/14,488	5,864,078	59.49	5,561,554	5,548	100.0/0.0	5,561,554/chromosome	95.2
								302,524/plasmid	102.7

## Data Availability

The genome assemblies and raw Oxford Nanopore sequencing reads described in this announcement have been deposited under BioProject PRJNA1389015. The annotated genome assemblies are available in GenBank, and the corresponding Sequence Read Archive (SRA) accessions are as follows: *Frigoribacterium* sp. CF331 (GCA_057787905.1, SRR36660841), *Glaciimonas* sp. OK215 (GCA_057787885.1, SRR36660840), *Dyella* sp. OK584 (GCF_057787755.1, SRR36660839), *Frondihabitans* sp. OV104 (GCA_057787745.1, SRR36660838), and *Shinella* sp. OV683 (GCA_057787725.1, SRR36660837). Genome annotations were generated using the NCBI Prokaryotic Genome Annotation Pipeline (PGAP). A KBase narrative containing GTDB assignments, annotations, and links to alternative assemblies and supplementary annotations generated during training and evaluation is publicly available at https://doi.org/10.25982/232191.29/3005254.
